# The Development of Neurological Damage in a Patient With Thrombotic Thrombocytopenic Purpura and Legionnaires’ Disease: A Case Report

**DOI:** 10.7759/cureus.42277

**Published:** 2023-07-21

**Authors:** Vincent Chen, Kanika Gulia, Christiane How-Volkman, John J Geraghty, Forshing Lui

**Affiliations:** 1 College of Medicine, California Northstate University College of Medicine, Elk Grove, USA; 2 Department of Neurology, Kaiser Permanente Roseville Medical Center, Roseville, USA; 3 Department of Clinical Sciences, California Northstate University College of Medicine, Elk Grove, USA

**Keywords:** brain infarction, legionella pneumophila, adamts13 protein, thrombotic thrombocytopenic purpura, legionnaires disease

## Abstract

This case report aims to highlight a rare and severe presentation of Legionnaires’ disease complicated by thrombotic thrombocytopenic purpura (TPP). The patient, a 75-year-old male with a history of COVID-19 infection, presented with bilateral pneumonia positive for *Legionella pneumophila*. He developed signs of TTP, cerebral hemorrhage, and renal failure. Despite treatment, the patient’s condition deteriorated, leading to flaccid paralysis, absent reflexes, and multiple brain hemorrhages. This case suggests a potential autoimmune mechanism for the neurological symptoms seen in this combination of Legionnaires’ disease and TTP. Thus, it would be worthwhile to further investigate and understand the relationship between these two conditions. Further research into underlying mechanisms will contribute to improving therapeutic approaches for this rare presentation. Additionally, the patient’s previous COVID-19 infection could have contributed to thrombotic complications due to its association with respiratory infections, warranting further investigation.

## Introduction

Thrombotic thrombocytopenic purpura (TTP) is an extremely rare blood disorder that affects 3-10 adults per million each year [1-3]. The exact etiology isn’t clear, but the disease has been associated with deficiencies in ADAMTS13, a metalloproteinase that cleaves von Willebrand factor polymers [4-8]. Without ADAMTS13, the polymers amass, resulting in the formation of microthrombi throughout the small vessels of the body [4-8]. Cellular injury and end-organ damage can occur as a consequence of ischemic blood flow.

This case report highlights a neuropathologic association between TTP and Legionnaires’ disease, a form of pneumonia caused by *Legionella* bacteria. The two diseases can produce similar neurological symptoms, though the pathophysiologies are distinct. In the rare instances when they are acquired in association, however, the pathogenesis of the symptoms becomes less clear.

The patient in this report presented to the hospital with bilateral pneumonia positive for *Legionella pneumophila* and quickly developed signs of TTP, cerebral hemorrhage, and renal failure. After 12 days of admission at the hospital, the patient expired.

## Case presentation

The patient is a 75-year-old male who presented to the hospital with a one-week history of shortness of breath that has worsened over the past three days with nausea and vomiting. Of note, the patient was infected with COVID-19 eight months earlier. He has a past medical history of hypertension, hyperlipidemia, coronary artery disease, and pituitary adenoma.

Upon admission, the patient was alert, awake, and able to provide a history. He denied any chest pains but had a non-productive cough. On physical examination, he had a blood pressure of 117/57 mmHg and was tachycardic, with a heart rate of 142 beats per minute. He was tachypneic and hypoxic as well, with a respiratory rate of 24 breaths per minute and a 77% oxygen saturation rate on room air. The patient was afebrile but appeared dehydrated.

The abdomen was soft, and the chest was clear to auscultation. X-ray images of the chest showed bilateral lung consolidation consistent with pneumonia. Sepsis was determined with admission lab values, which showed an elevated white blood cell count of 24.1 K/uL and an abnormally high lactate level of 3 mmol/L. The patient also had low levels of red blood cells (3.85 M/uL) and hemoglobin (11.6g/dL), as well as low hematocrit (33.9 %) values. The patient showed elevated blood urea nitrogen (33 mg/dL) and creatinine (2.08 mg/dL), consistent with renal dysfunction. The platelet count was normal (296 K/uL). The patient was then placed on a BiPAP machine by the hospital team.

On Day 2, the patient deteriorated. WBC count rose to 27.4 K/uL. Procalcitonin levels were found to be greatly elevated at 11.36 ng/mL, indicating a severe bacterial infection. The patient was found to be positive for *Legionella* antigen. The patient had to be intubated, and ceftriaxone, doxycycline, and cefepime were empirically administered. Lovenox was started for deep vein thrombosis prophylaxis as well.

On the same day, the RBC count dropped (3.62 M/uL), as did hemoglobin (10.8 g/dL) and hematocrit (31.9%). Red cell morphology study showed Burr cells, anisocytosis, poikilocytosis, polychromasia, and large platelets, indicating anemia or another blood disorder. However, blood tests showed that the patient was negative for anti-nuclear antibodies and antineutrophil cytoplasmic antibodies. Serotonin release assay also returned negative.

The following day, WBC levels climbed to 48.2 K/uL, while hematocrit dropped to 29.4 %. Vancomycin was added. Platelet levels remained within the normal range at 307 K/uL.

By the end of Day 4, the patient’s platelet levels had dropped to 154 K/uL, and Lovenox was changed to heparin 5000 units twice daily. The WBC remained elevated at 33.5 K/uL. Levaquin was added.

On Day 5, the patient’s platelet levels dropped further to 123 K/uL. WBC count remained elevated. The next day, platelet levels decreased further still, to 104 K/uL, and heparin was discontinued. Renal failure occurred on Day 7 and hemodialysis was begun.

On Day 8, the patient developed prominent purpura and ecchymosis. The patient’s platelets dropped to 58 K/uL, and the IgG PF4 antibody was found to be negative. From Day 8 onwards, the platelet count failed to rise above 100 K/uL. The complete results of the blood tests can be found in Table 1.

**Table 1:** Blood test results over the course of admission until passing. WBC counts remained high throughout the patient’s admission consistent with their Legionella infection. Although RBC remained normocytic, RBC counts, HGB, HCT, and platelet counts gradually depleted over the course of two weeks suggestive of TTP. WBC: white blood cell, RBC: red blood cell, HGB: hemoglobin, HCT: hematocrit, MCV: mean corpuscular volume, RDW: red cell distribution width, NRBC: nucleated red blood cells, PLT: platelet, L: low, H: high, A: adjusted

Component	1/13/2022 (Day 1)	1/14/2022 (Day 2)	1/15/2022 (Day 3)	1/16/2022 (Day 4)	1/17/2022 (Day 5)	Reference values
WBC (K/uL)	24.1 (H)	27.4 (H)	48.2 (A)	33.5 (A)	32.5 (A)	4.5-11.0
RBC (x10^6^/uL)	3.85 (L)	3.62 (L)	3.29 (L)	2.85 (L)	2.84 (L)	4.5-5.9
HGB (g/dL)	11.6 (L)	10.8 (L)	9.9 (L)	8.4 (L)	8.5 (L)	14-17.5
HCT (%)	33.9 (L)	31.9 (L)	29.4 (L)	26.2 (L)	26.0 (L)	41.5-50.4%
MCV (fl)	88	88	89	92	92	80-100
RDW (fl)	15.7	15.8	16.2	16.8 (H)	16.9 (H)	12-15
NRBC (%)	0	0	0	0	0	0
PLT (K/uL)	296	261	318	154	123 (L)	150-450
WBC (K/uL)	37.8 (A)	36.2 (A)	27.8 (H)	46.4 (A)	35.5 (A)	39.0 (A)
RBC (x10^6^/uL)	3.09 (L)	3.10 (L)	2.86 (L)	3.49 (L)	2.85 (L)	2.93 (L)
HGB (g/dL)	9.2 (L)	9.3 (L)	8.4 (L)	10.5 (L)	8.6 (L)	8.9 (L)
HCT (%)	27.4 (L)	27.9 (L)	24.8 (L)	31.4 (L)	25.8 (L)	26.6 (L)
MCV (fl)	89	90	87	90	90	91
RDW (fl)	17.0 (H)	17.6 (H)	16.3	16.0	16.0	16.0
NRBC (%)	0	0	0	1 (H)	1 (H)	0
PLT(K/uL)	104 (L)	58 (L)	28 (A)	97 (L)	62 (L)	57 (L)

**Figure 1:**
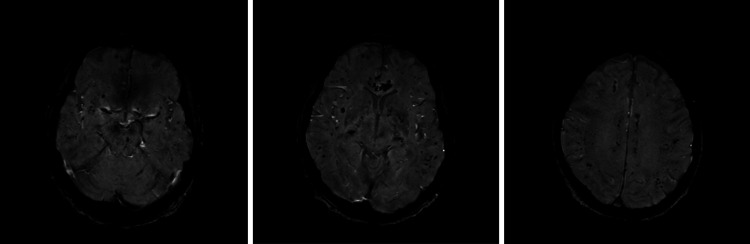
CT scan demonstrating the development of multiple small infarcts in a patient with respiratory and hematologic comorbidity factors.

Neurology was asked to evaluate the patient on Day 11. Examination found flaccid paralysis and absent reflexes in all four limbs. Corneal blink reflexes were absent, and 2 mm pupils were found to be non-reactive. The patient was unresponsive to sedatives and showed no response to plantar stimulation. CT scan showed multiple small hemorrhages (Figure 1). After failure to improve, the decision was made to make the patient comfortable. He expired with his family by the bedside.

## Discussion

Legionnaires' disease has been well characterized in the pulmonary system, but its mechanism in the renal, hepatic, and neurologic systems is still under investigation. While severe cases of septic pneumonia can be indicated by cultures of *Legionella pneumophila* in the respiratory tract, dysfunctions in other organ systems are not associated with pathologic changes or isolation of the bacterium [9,10]. Circulating protein toxins have been implicated, as they have autoimmunological mechanisms [10-14].

In one case of Legionnaires' disease, a patient developed hematuria, pyuria, and cylinduria without depression in complement levels or signs of vasculitis [10]. The authors hypothesized glomerulonephritis occurred due to a circulating toxin or an immune-mediated disease [10].

Similar mechanisms may underlie the neurological manifestations of Legionnaires' disease. Most often, they consist of headaches and non-specific changes in levels of consciousness, along with confusion, memory deficits, and, rarely, visual hallucinations [9,14-16]. These symptoms may be attributable to hypoxia, uremia, and metabolic changes, but they are less likely to explain more critical neurological presentations [10]. One patient reportedly showed severe weakness in the neck, shoulder, and pelvic girdle muscles, with absent deep tendon reflexes and a positive Romberg sign [10]. Another case report described two patients with bradyphrenia, apraxia, and tactile extinction of the left arm and fluctuating levels of consciousness [13]. Similarly, our patient showed flaccid paralysis, absent reflexes in all four limbs, and eventually entered into a coma. As hypothesized by other authors, an immune-mediated mechanism is very likely involved in the pathogenesis of these neurological symptoms [10,13].

Less clear, however, is the relationship between Legionnaires' disease and TTP, a rare blood disorder classically characterized by a pentad of microangiopathic hemolytic anemia, thrombocytopenia, neurological changes, fever, and renal dysfunction [6,8]. To the best of our knowledge, there have only been three reports linking Legionnaires' disease and TTP; in all three cases, TTP complicated Legionnaires’ disease and required adjustments to treatment programs [6,17,18]. A better understanding of the two pathologies’ relationship will allow for earlier diagnoses and more targeted treatments.

Our case is the first to describe the neurological damage inflicted by the unique combination of *Legionella* and TTP. After acquiring severe septic pneumonia positive for Legionnaires' disease, our patient showed deteriorating platelet levels, renal dysfunction, and abnormal red blood cell morphologies, all pointing towards TTP. Notably, CT scans showed multiple small infarcts in the brain of the patient. The patient also presented with nonreactive pupils, absent blink reflexes, and lack of plantar response, in addition to flaccid paralysis and areflexia in all four limbs. In contrast to most cases of Legionnaires' disease, the neurological symptoms failed to resolve and the patient expired.

We hypothesize that an autoimmune process underlies the patient’s presentation. The unique severity of his neurological symptoms appears to be the result of an exacerbating relationship between Legionnaires' disease and TTP. In the two aforementioned cases that linked Legionnaires' and TTP, the authors suggested that antibodies developed against *Legionella* cross-reacted with ADAMTS-13, leading to microthrombi and platelet consumption [6,17]. This would be consistent with one of the proposed mechanisms by which Legionnaires' disease precipitates multisystem dysfunctions. In our patient, it is possible that TTP was similarly acquired through an autoimmune process, and intracerebral hemorrhage followed, as has been reported in rare instances of TTP [1].

This report demonstrates that a better understanding of the relationship between TTP and Legionnaires' disease is needed for prompt diagnosis and execution of an appropriate treatment plan. Though rare, infarctional damage as a result of *Legionella* is a critical diagnosis.

It should be noted that the patient contracted COVID-19 eight months before acquiring Legionnaires' disease. COVID-19 has been associated with TTP and other thrombolytic syndromes [19]. However, these complications have presented only in critically ill patients and have not been reported in those who have recovered from COVID-19 [19].

## Conclusions

Though the mechanisms of TTP and Legionnaires' disease are individually well understood, the pathogenesis of their concurrent presentation remains unclear. Further studies are required to explore if there is an underlying autoimmune process or if there are other genetic factors that may predispose patients to this unique presentation.
